# Addressing Balance Issues in Diabetic Foot Ulcer Patients: Offloading, Mobility, and Fall Prevention

**DOI:** 10.7759/cureus.92803

**Published:** 2025-09-20

**Authors:** Elangkumaran V Manoharan, David Hughes, Hamzeh AL-Arqan

**Affiliations:** 1 Diabetes and Endocrinology, Blackpool Teaching Hospital, Blackpool, GBR; 2 Diabetes and Endocrinology, University Hospitals of Derby and Burton NHS Foundation Trust, Derby, GBR

**Keywords:** balance issues, diabetic foot ulcers, diabetic neuropathy, fall prevention, fall risk, mobility assessment, mobility limitations, physical therapy, quality of life

## Abstract

Diabetic foot ulcers (DFUs) are a prevalent and debilitating complication of diabetes, often resulting in extended recovery periods and diminished quality of life. Effective pressure offloading is vital for promoting ulcer healing, using devices such as total contact casts, removable walkers, and specialized footwear. However, while these tools are designed to alleviate pressure on the affected areas, they can unintentionally affect balance and walking patterns, potentially raising the risk of falls and limiting mobility. This review explores how different offloading methods impact postural control and fall risk in people with DFUs. It assesses the mechanical changes these devices introduce, reviews current techniques for measuring balance and mobility, and discusses supportive interventions like physiotherapy, gait rehabilitation, and fall prevention strategies. In addition, the review considers the psychological and social challenges, such as anxiety, reduced confidence, and social withdrawal, that may affect recovery. Gaps in existing research are identified, with recommendations for future work aimed at balancing effective ulcer treatment with the need to maintain patient safety and independence. Addressing both physical and emotional factors is key to improving outcomes and preserving quality of life for those living with DFUs.

## Introduction and background

Diabetic foot ulcers (DFUs) are a common and serious complication of diabetes, particularly in older adults. Beyond their impact on wound healing, DFUs significantly impair patients’ mobility and balance, resulting in increased fall risk and reduced quality of life [1]. Addressing balance impairments in individuals with DFUs is essential, as falls can severely hinder rehabilitation, prolong recovery, and increase morbidity [2].

Offloading devices, such as total contact casts and removable cast walkers, are essential components of DFU management, as they redistribute plantar pressure to promote ulcer healing. However, these devices often disrupt natural gait patterns and compromise postural stability. This disruption may lead to adverse outcomes such as musculoskeletal pain, falls, and fractures, further limiting mobility and delaying recovery. Additionally, many individuals with DFUs suffer from comorbid conditions such as peripheral neuropathy, which impairs proprioception and further exacerbates balance issues [3].

The resulting decline in mobility can contribute to decreased physical activity, social isolation, and psychological distress, all of which negatively impact overall health and well-being. Therefore, managing balance disturbances in DFU patients requires a comprehensive, multidisciplinary approach that combines wound care with individualized fall prevention strategies [4].

Healthcare professionals must consider a range of patient-specific factors, including physical limitations, comorbid conditions, and psychosocial stressors, when developing management plans. Interventions such as physical therapy, gait training, and personalized fall prevention programs play a critical role in restoring mobility and minimizing fall risk [5]. Furthermore, addressing emotional and social concerns, such as fear of falling, frustration, and isolation, can improve treatment adherence and enhance clinical outcomes.

By incorporating both physical and psychological assessments into routine care, healthcare providers can improve the overall quality of life for individuals living with DFUs [6]. This review aims to provide a comprehensive overview of the challenges associated with balance and mobility in DFU patients and to explore evidence-based strategies that can mitigate these issues and improve patient outcomes.

## Review

Role of offloading and pressure distribution 

Offloading is a cornerstone of DFU management, aimed at redistributing plantar pressure away from ulcerated areas to facilitate wound healing [7]. A variety of offloading modalities are employed, including custom-made therapeutic footwear, total contact casts (TCCs), removable cast walkers (RCWs), and temporary postoperative padded open-toe shoes. These devices help alleviate localized pressure by redistributing mechanical loads across the foot and lower leg (Table 1) [8,9].

**Table 1:** Types of offloading devices used in diabetic foot ulcer (DFU) management The table summarizes the principal offloading devices used in the management of DFUs. Offloading devices are designed to redistribute plantar pressure, reduce mechanical stress at ulcer sites, and facilitate wound healing. The table provides an overview of device type, mechanism of action, clinical advantages, and impact on patient mobility. Citations reference systematic reviews, guidelines, and clinical studies that evaluated the efficacy and limitations of each device. This information enables clinicians and researchers to interpret comparative effectiveness and potential barriers to adherence.

Offloading device	Description	Advantages	Impact of mobility
Total contact cast (TCC)	A rigid, padded cast that encases the foot and lower leg, redistributing pressure across the foot surface.	Provides maximum offloading and reduces weight-bearing directly on the ulcer site.	Limits natural gait and ankle movement, reduces mobility, and requires adaptation to walking [8,10]
Removable cast walker (RCW)	A boot-like device that can be removed, offering support and offloading for DFU patients.	Easier to remove and adjust; provides adequate offloading.	Offers flexibility, but bulkiness can affect walking efficiency, causing slower and less efficient movement [13]
Moulded insoles	Specialized insoles designed to redistribute pressure across the foot.	Easy to use with regular shoes and lightweight.	Improves comfort and offloading, but mobility may still be impaired due to pressure on ulcerated areas [14]
Ankle-foot orthosis (AFO)	A brace that supports the ankle joint, often used for neurological conditions and to correct gait.	Lightweight and less bulky than TCC; can be worn with regular shoes.	Allows for some mobility, though less effective in offloading compared to rigid devices; may not significantly improve gait [15]
Temporary padded postoperative shoe/sandal	A rigid-soled, padded fabric footwear with adjustable straps.	Provides plantar offloading and can be adjusted to accommodate dressings.	Rigid base limits natural gait and requires adaptation to walking [8]

TCCs and standard air walker boots provide comprehensive support by encasing the foot and lower leg, promoting even pressure distribution. RCWs have gained popularity due to their ease of use, as they can be removed for dressing changes and wound inspection, offering greater flexibility for both patients and clinicians [10]. However, despite their clinical utility, these devices can significantly disrupt gait mechanics and balance. By restricting natural foot and ankle movements and adding approximately 1-2 kg of weight, they may contribute to gait asymmetry and postural instability [11].

Patients often report reduced walking speed, decreased endurance, and difficulty performing daily activities. These challenges are particularly pronounced in older adults, where age-related muscle loss, or sarcopenia, may further impair the ability to adapt to the added weight and altered biomechanics of offloading devices. Despite these drawbacks, effective offloading remains essential in DFU care. It reduces mechanical stress on vulnerable tissues, prevents further injury, and enhances the wound healing process, ultimately decreasing the risk of infection and serious complications, including lower-limb amputation [12]. 

Fall risk in DFU patients

Patients with DFUs are significantly more prone to falls than the general population, with particularly high risk among those with severe peripheral neuropathy and diabetic retinopathy. The dual burden of physical limitations from the ulcer itself and mobility restrictions from offloading devices renders this population especially vulnerable to instability and injury [16]. Several interrelated factors contribute to this elevated fall risk.

Neuropathy 

Peripheral neuropathy, a common complication of diabetes, diminishes the ability to perceive pressure, temperature, and surface irregularities, which are all essential for maintaining balance [17]. The absence of these sensory cues hinders patients’ ability to detect environmental hazards such as uneven ground or obstacles, thereby increasing the likelihood of slips, trips, and falls. Moreover, proprioceptive deficits impair the patient’s awareness of limb position and movement, leading to poor coordination. When compounded by the altered biomechanics of offloading footwear, these deficits significantly heighten the risk of falls [18]. 

Impaired Vision 

Visual impairments, such as those caused by diabetic retinopathy, further contribute to instability by reducing depth perception, peripheral vision, and contrast sensitivity. These deficits make it more difficult to navigate unfamiliar or cluttered environments, detect changes in terrain, and judge distances accurately, all of which are critical for safe ambulation. 

Offloading Footwear 

Although offloading devices are essential for ulcer healing, they are not without drawbacks. These devices are typically designed with rigid bases or shell-like exteriors to restrict foot and ankle movement. While many patients eventually adapt to these devices, they often induce abnormal gait patterns and impair balance, especially during tasks such as stair climbing, turning, or walking on uneven surfaces. These altered gait mechanics increase the likelihood of stumbling and falling [19]. 

Weakness and Deconditioning 

Many patients with DFUs, particularly older adults, experience muscle weakness and general deconditioning because of reduced physical activity, prolonged immobility, or chronic illness. Sarcopenia, the age-related loss of muscle mass, can further impair strength and balance. Lower limb weakness limits the ability to bear weight effectively or recover from perturbations in posture, significantly increasing the risk of falls [20].

Consequences of falls 

Falls in patients with DFUs can result in serious physical and psychological consequences, further complicating clinical outcomes and quality of life. Physically, common injuries include fractures, sprains, and soft tissue damage, which are particularly concerning in this population due to impaired healing capacity, often linked to microvascular dysfunction and peripheral arterial disease [21]. Even minor trauma can lead to significant complications, particularly in patients with peripheral neuropathy who may fail to detect injuries promptly due to sensory loss. This delayed recognition can increase the risk of infection, delayed wound healing, and even ulcer recurrence [22]. 

Beyond the physical effects, falls can have profound psychological consequences. Many patients develop a fear of falling, which can contribute to anxiety, depression, reduced self-confidence, and social withdrawal. This fear often results in decreased physical activity, which further exacerbates muscle deconditioning and balance impairment-creating a vicious cycle that increases the risk of subsequent falls [23]. 

Effective management of fall-related consequences requires not only physical rehabilitation and wound care but also attention to the psychological well-being of the patient. A multidisciplinary approach that addresses both the physical and emotional impact of falls is essential to break this cycle and support recovery. 

Mobility limitations with offloading footwear 

Impact on Gait and Function 

Offloading footwear, including TCCs and RCWs, plays a critical role in DFU management by redistributing plantar pressure and promoting wound healing. However, these devices often restrict ankle mobility and disrupt natural gait mechanics. Patients commonly adapt by shortening their stride length, widening their stance, and reducing walking speed. These compensatory changes may lead to decreased walking efficiency, increased fatigue, and secondary discomfort in proximal joints such as the hips and knees. Over time, such altered biomechanics can contribute to muscular deconditioning and further elevate the risk of falls, ultimately limiting both mobility and independence in daily activities [24]. 

Patient-Reported Challenges 

Many patients report significant challenges while using offloading devices due to their rigidity, bulkiness, and incompatibility with normal clothing and footwear. These characteristics often hinder participation in routine activities such as shopping, attending appointments, or engaging in social interactions. As a result, these barriers can reduce adherence to prescribed offloading regimens, compromising both ulcer healing and overall clinical outcomes [25]. 

Limitations in Functional Performance 

The mechanical constraints imposed by offloading devices can severely impact functional performance. Tasks requiring dynamic foot and ankle movement, such as stair climbing, prolonged walking, or standing for extended periods, are often difficult or impossible for patients. This limitation not only reduces physical activity levels and quality of life but also increases dependency on caregivers and support systems [26]. 

Assessment tools for balance and mobility 

A variety of standardized tools are available to assess balance and mobility in patients, including those with DFUs. While some of these tools are primarily used in research settings, many can be effectively applied in outpatient clinics to evaluate fall risk and functional capacity. Accurate assessment supports clinical decision-making and guides intervention planning. 

Timed Up and Go (TUG) Test 

The TUG test measures the time taken for a patient to stand up from a seated position, walk three meters, turn around, return to the chair, and sit down. It is a simple yet effective assessment of functional mobility and dynamic balance. Prolonged completion times are associated with impaired mobility and an increased risk of falls [27]. 

Berg Balance Scale (BBS) 

The BBS evaluates static and dynamic balance using 14 functional tasks, such as standing up from a seated position, reaching forward, and turning. Each task is scored on a scale, with lower overall scores indicating poorer balance and a greater risk of falling [28]. 

Dynamic Gait Index (DGI) 

The DGI assesses a patient’s ability to modify gait in response to various external challenges, such as changes in speed, walking around obstacles, or turning the head while walking. It is particularly useful in identifying gait and balance impairments in dynamic, real-world scenarios [29]. 

Functional Reach Test 

This test evaluates the stability of a patient’s standing balance by measuring how far they can reach forward without losing balance or taking a step. A shorter reach distance is correlated with reduced balance control and a higher fall risk [30]. 

Specific considerations for DFU patients 

The complex interplay of peripheral neuropathy, impaired circulation, foot deformities, and the use of offloading devices necessitates a tailored assessment approach for patients with DFUs [31]. A multidisciplinary team, including podiatrists, physiotherapists, and other healthcare professionals, is often essential to provide comprehensive evaluation and individualized care planning [32].

Podiatrists play a key role in assessing ulcer status, ensuring appropriate offloading device fit and function, and evaluating the impact of these devices on foot mechanics and mobility [33]. Physiotherapists contribute by assessing balance, muscle strength, and gait mechanics, considering the functional limitations imposed by the ulcer and offloading footwear. This collaborative approach is critical to thoroughly evaluating and addressing the multifaceted issues of balance, mobility, and fall risk in this population [34].

By integrating the expertise of various disciplines, healthcare providers can develop personalized care plans that address both ulcer healing and overall functional mobility. Continuous monitoring and regular reassessment of balance and mobility are necessary to track patient progress and adjust treatment strategies, especially when clinical conditions evolve or deteriorate [35].

Interventions to improve balance 

Physical Therapy and Gait Training 

Physical therapy is essential for enhancing balance and mobility in patients with DFUs, particularly those limited by offloading devices. Gait training focuses on restoring more natural walking patterns, which are often disrupted by rigid devices such as TCCs and RCWs [36]. Through targeted exercises, patients learn to compensate for restricted ankle movement by improving posture, foot placement, and step coordination. Strengthening key lower limb muscles, including the calves, quadriceps, hamstrings, and core muscles, is also critical, as these groups support postural stability and help patients recover from balance disturbances. Balance training, involving activities such as walking on uneven surfaces, use of balance boards, or single-leg standing, further enhances proprioception, coordination, and body awareness [37,38]. Collectively, these therapies reduce fall risk and improve functional mobility, enabling patients to better tolerate and manage their offloading devices. 

Footwear and Orthotics

Custom therapeutic footwear and orthotic insoles play a vital role in long-term management by preventing re-ulceration and supporting balance [39]. These devices should provide adequate cushioning, support, and stability while maintaining proper foot and ankle alignment. Comfort and ease of use are paramount to encourage consistent adherence. Custom orthotics redistribute plantar pressure and reduce focal stress points, thereby minimizing the risk of new ulcer formation and enhancing overall foot function [40]. 

Fall Prevention Programs 

Fall prevention programs should be integrated with environmental modifications to enhance patient safety. Home safety interventions, including removal of tripping hazards, securing loose carpets, improving lighting, and installing grab bars or handrails, can significantly reduce the risk of falls in individuals with impaired mobility [41,42]. These combined strategies are essential to creating a safer living environment and promoting independence. 

Assistive Devices 

When appropriate, assistive devices such as canes, walkers, or crutches may be prescribed to improve balance and reduce fall risk [43]. These tools provide additional support for patients experiencing muscle weakness, poor balance, or during recovery from ulcers or injuries. Proper selection and fitting of assistive devices are critical to ensure compatibility with offloading footwear, prevent additional strain, and maximize safety. Devices should be lightweight and adjusted to the correct height for optimal function. When used correctly, assistive devices can enhance mobility, increase confidence, and reduce falls [44]. 

Psychosocial considerations 

The physical limitations imposed by DFUs and the use of offloading devices can profoundly affect patients’ mental health and social well-being. The chronic nature of DFUs, combined with prolonged treatment regimens, often leads to feelings of frustration, helplessness, and emotional distress, particularly when offloading devices interfere with routine daily activities [45]. Many patients experience a diminished sense of control, which can adversely impact their psychological well-being [46]. 

Specialized offloading footwear may also contribute to feelings of embarrassment or stigma, reducing social confidence and prompting withdrawal from social interactions. This effect is especially pronounced among younger patients, who may prefer to wear fashionable shoes instead of prescribed therapeutic footwear, resulting in reduced adherence to treatment and delayed healing [47]. 

Healthcare providers must address these emotional and social challenges by offering psychological support, counselling, and strategies that help patients maintain self-esteem and quality of life throughout their treatment journey. Integrating psychosocial care into the management of DFUs is crucial for optimizing adherence and improving overall outcomes [48]. 

Research gaps and future directions 

Effective management of fall risk and adherence to offloading device use in patients with DFUs remains limited by significant gaps in knowledge. While offloading devices are designed to redistribute pressure and promote ulcer healing, their impact on gait mechanics, mobility, and fall risk varies widely across different devices and individual patients. This variability, coupled with the frailty and multiple comorbidities often present in DFU patients, underscores the need for comparative studies aimed at developing evidence-based guidelines tailored to specific patient subgroups based on factors such as comorbid conditions, mobility status, self-efficacy, and fall risk [49]. 

Moreover, there is a scarcity of long-term data examining the effects of offloading devices on related comorbidities, such as heart failure, osteoarthritis, and obesity, as well as on mobility, balance, psychological well-being, social engagement, and overall quality of life. Most existing studies focus on short-term outcomes, limiting understanding of sustained benefits or harms [50]. 

Additionally, personalized fall prevention interventions remain underutilized in clinical practice despite their potential importance for improving long-term outcomes in DFU patients, particularly those challenged by neuropathy, muscle weakness, and the burden of heavy offloading devices [51]. 

Future research should prioritize investigating the clinical efficacy and cost-effectiveness of diverse offloading strategies and behavioural support programs. Such efforts will enable healthcare professionals to adopt personalized, evidence-based approaches that effectively reduce fall risk, enhance mobility, and improve the overall quality of life for patients living with DFUs [52]. 

## Conclusions

DFUs are a common and complex condition that requires coordinated care across multiple healthcare disciplines. While offloading devices play a vital role in healing ulcers, it is important to balance their use with maintaining patients’ mobility, gait, and stability. Care plans should be customized to address each patient’s physical abilities as well as emotional and social needs. Supporting mental health and reducing feelings of isolation are key, since these factors can influence how well patients follow treatment and their overall well-being. Continued research is needed to find offloading methods that not only aid healing but also preserve long-term mobility and quality of life. 
